# Persistent tissue regeneration and transforming growth factor-β induced fibrosis in the masseter muscle of *mdx*^*5Cv*^ mice

**DOI:** 10.1038/s41598-025-17154-3

**Published:** 2025-09-29

**Authors:** Aouatef Ait-Lounis, Laurence A. Neff, Stavros Kiliaridis, Bernhard Wehrle-Haller, Olivier M. Dorchies, Gregory S. Antonarakis

**Affiliations:** 1 Division of Orthodontics, University Clinics of Dental Medicine, Faculty of Medicine, University of Geneva, Geneva, Switzerland; 2 Pharmaceutical Biochemistry/Chemistry Group, School of Pharmaceutical Sciences, University of Geneva, Geneva, Switzerland; 3 Institute of Pharmaceutical Sciences of Western Switzerland, University of Geneva, Geneva, Switzerland; 4 Laboratory of Fundamental and Applied Bioenergetics, INSERM U1055, Université Grenoble Alpes, Grenoble, France; 5 Department of Orthodontics and Dentofacial Orthopaedics, Dental School, Medical Faculty, University of Bern, Bern, Switzerland; 6 Department of Cell Physiology and Metabolism, Faculty of Medicine, University of Geneva, Geneva, Switzerland

**Keywords:** Duchenne muscular dystrophy, DMD, *mdx*^*5Cv*^, Masseter muscles, Fibrosis, FAPs, Extracellular matrix, TGF-β signalling, Cell biology, Diseases, Pathogenesis

## Abstract

Clinical observational studies have shown that patients with Duchenne muscular dystrophy (DMD) often develop orofacial dysfunction. However, most DMD mouse model studies have focused on limb and respiratory muscles, showing an extensive wave of muscle necrosis-regeneration in juveniles followed by a low-intensity chronic disease in adults. In the present study, we investigated the impact of DMD on the masseter muscles in mice and observed persistence and progression of the conditions at least until 12 months of age. Masseter and limb muscles from *mdx*^*5Cv*^ mice aged 3, 6, and 12 months were compared with those from control mice (C57BL/6 J background), measuring levels of necrosis, regeneration, inflammation, and fibrosis. In addition, mRNA expression of markers associated with fibrosis and transforming growth factor-beta (TGF-β) signalling were examined. Our findings revealed that regeneration and inflammation were increased in dystrophic masseter muscles at 3 months of age and persisted to older ages. Fibrosis was more pronounced in the dystrophic masseter muscles at 6 months of age and revealed an increase of the fibro-adipogenic progenitor cell population. Notably, we found elevated deposition of fibronectin and TGF-β in fibrotic foci of the dystrophic masseter muscles. Increased TGF-β signalling was confirmed by a significant increase in nuclear localization of phosphorylated SMAD2 in dystrophic masseter and limb muscles. Our results suggest that masseter muscles may exhibit more sustained dystrophic damage than locomotor muscles. We speculate that any therapeutic developed for DMD may be of benefit to orofacial tissues, although their efficacy remain to be established.

**Supplementary Information:**

The online version contains supplementary material available at 10.1038/s41598-025-17154-3.

## Introduction

Duchenne muscular dystrophy (DMD) is an X-linked muscular disorder that primarily affects boys. With an estimated incidence of 1/3500–1/9000 male births, DMD is one of the most prevalent muscular dystrophies in childhood^1^. DMD results from mutations in the *DMD* gene that lead to the absence of functional dystrophin, causing impairment of myofiber homeostasis, loss of membrane integrity, and continuous degeneration of skeletal muscles^2^. The repetitive muscle injuries lead to muscle fibre death, chronic inflammation, and severe fibrosis, and finally progressive muscle wasting, which is among the most distinguishing characteristics of DMD^3^. Ultimately, this leads to loss of ambulation in the teenage years and premature death usually before the age of 40, occurring secondary to cardiac or respiratory failure^4^.

Almost all preclinical research, clinical assessment of therapeutics, and daily care management is focused on preserving locomotor muscles and cardiac function. In fact, mastication and craniofacial concerns are often ignored in current DMD guidelines on diagnosis and patient management. No indication is given concerning follow-up and evaluation by a dental or orthodontic professional, which is reflected in the lack of such mention in recent guidelines of the DMD care consideration working group^5^.

However, it is well established that patients with DMD show altered structure and impaired function of head and neck muscles, associated with dentofacial defects and malocclusion, which deteriorate with age, due to both progressive muscular alterations and aberrant dentofacial growth^6–8^. These alterations include reduced mouth opening, reduced bite force, impaired quality of the structure of the masseter muscles, enlargement of the tongue (macroglossia), high prevalence of malocclusions associated with posterior crossbites and anterior and lateral open bites, a tendency toward mandibular prognathism, wider dental arches with an excess of space, dental compensations, and reduced lip force (although relatively stable during growth). Muscular alterations can also complicate oral health issues and create dental care challenges for individuals with DMD^9^.

However, the mechanism by which these changes come about, as well as the muscle-skeleton crosstalk, in boys suffering from DMD has not been sufficiently elucidated. The use of a DMD mouse model is therefore warranted. Although some studies have investigated masticatory muscles (principally the masseter muscles) in mice with DMD (*mdx* models)^10–12^, no study has focused on the progression of the fibrosis in the masticatory muscles. Thus, the better characterization of the fibrotic progression in these muscles may provide key elements to understanding the pathological mechanisms of DMD.

Fibrosis is defined as pathological wound healing with an abnormal deposition of extracellular matrix (ECM) resulting in the replacement of functional tissue by fibrotic connective tissue, which leads to an alteration of tissue and organ homeostasis^13^. Fibrosis involves the alterations of intracellular and secreted extracellular soluble effectors, including pro- and anti-inflammatory cytokines, growth factors, and ECM remodelers such as transforming growth factor-beta (TGF-β), and collagens^14^. The main pathway sustaining this excessive pro-fibrotic response is the TGF-β pathway, which is overactivated in DMD^15,16^. Bernasconi et al. reported that the mRNA expression of TGF-β1 in both plasma and muscle was markedly increased in patients with DMD and the levels of TGF-β1 correlated with fibrosis^17^. TGF-β is expressed together with the latency-associated peptide (LAP), rendering TGF-β inactive by masking the receptor binding sites of the mature cytokine. This latent TGF-β-LAP complex is stored in the ECM of skeletal muscle and requires mechanical forces to be activated^18^. Precisely localised activation of TGF-β is essential to maintain cell function and homeostasis, while discontinuity of the temporal and spatial activation of TGF-β leads to skeletal complications, including myopathies, and more common musculoskeletal disorders such as DMD.

One way of TGF-β activation involves heteromeric cell-surface receptors of the integrin family, which are composed of *α* and *β* subunits. TGF-β activation during myofibroblast contraction requires the binding of integrin *α*v*β*6 to an arginyl–glycyl–aspartic acid (RGD) sequence in the LAP-prodomain of TGF-β. In response to this mechanically induced conformational change, the mature TGF-β cytokine is released^19^, potentially causing the fibrotic response in the DMD muscle. Specifically, one may postulate that activation of TGF-β via integrin *α*v*β*6 in masseter muscles is causing the pathogenesis seen in DMD.

The aim of this paper was therefore to analyse the histopathological changes and to characterize the different cell types, ECM proteins and TGF-β signalling pathways in masseter muscles of dystrophic mice at different ages corresponding to different stages of disease severity.

## Results

### Masseter muscles of ***mdx***^***5Cv***^ mice exhibit increased markers of dystrophic pathology

Dystrophic pathology in the diaphragm and lower limb muscles of *mdx* and the phenotypically similar *mdx*^*5Cv*^ mice has been intensively characterized previously by us and by others^20,21^.

**Fig. 1:**
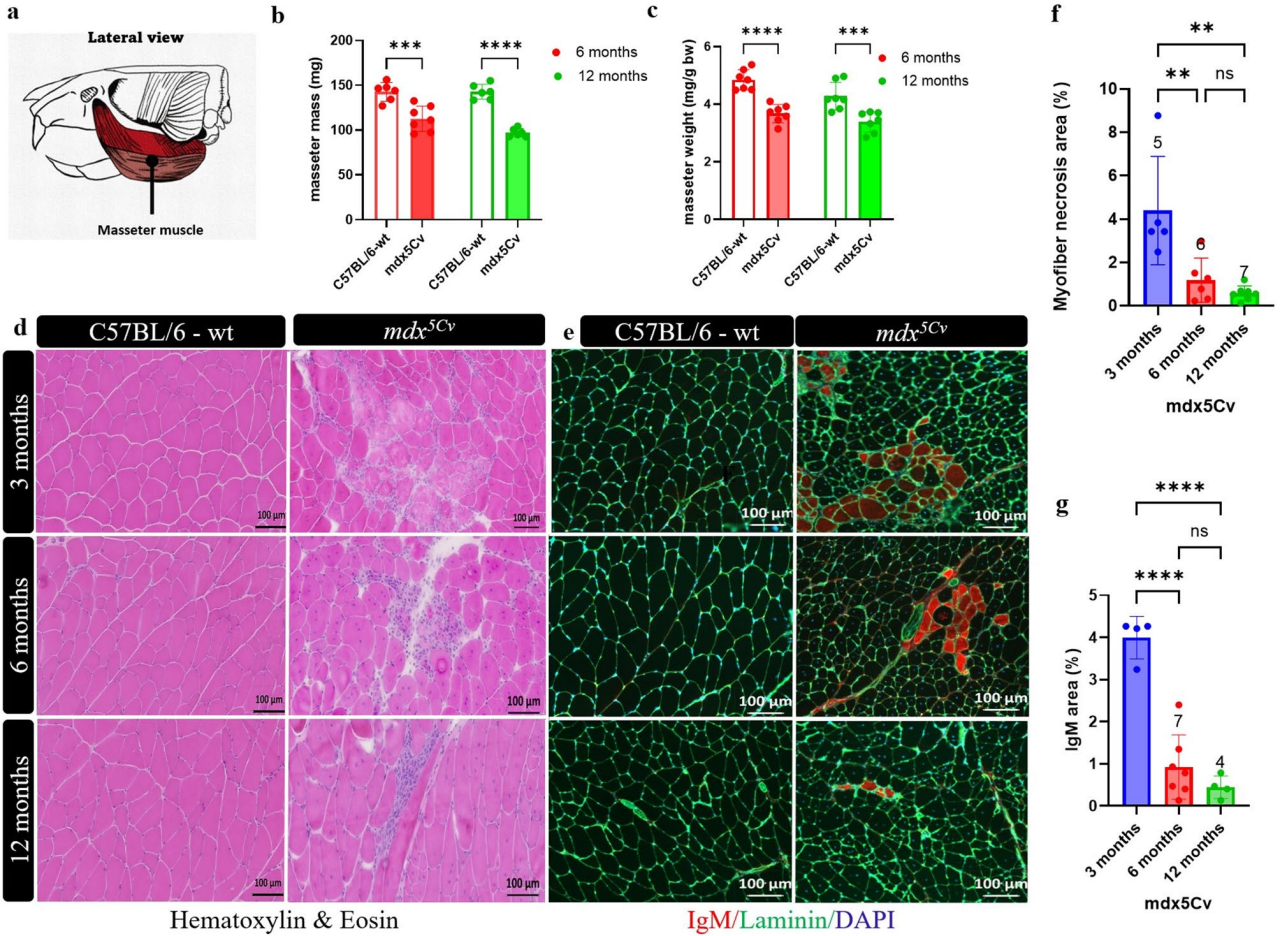
Myonecrosis and IgM infiltration are increased in masseter muscles of *mdx*^*5Cv*^ mice. Anatomical illustration of masseter muscle used in this study (**a**). The masseter muscle mass, from 6- and 12-month-old male *mdx*^*5Cv*^ mice and wild-type (wt) control littermates (*n* = 7–9 muscles) (**b**). The masseter muscle weight, normalized to body weight from 6- and 12-month-old male *mdx*^*5Cv*^ mice and wt littermates (*n* = 7–9 muscles) (**c**). Representative haematoxylin–eosin-stained sections of masseter muscles from 3-, 6- and 12-month-old wt and *mdx*^*5Cv*^ mutant mice (n = 5–7 mice/genotype) (**d**). Masseter muscles were immunostained for IgM (red), laminin (green), and DAPI (blue) (**e**). Sections were quantified to determine the percentage of total necrotic myofibers (infiltrating inflammatory cells area plus degenerated myofibers with fragmented sarcoplasm area/total muscle area) (**f**) and only IgM infiltrated muscle fibres (**g**). Data are presented as mean ± SD. Statistical analysis usied a two-way ANOVA with a post-hoc Šídák's multiple comparisons (**a**) and one-way ANOVA with a post-hoc Tukey’s test (**d**,**e**). *ns* not significant, *p < 0.05, **p < 0.01, ***p < 0.001, ****p < 0.0001.

These strains exhibit, similar to the classical *mdx* mouse model^22^, a mild and progressive dystrophic muscle phenotype with myofiber degeneration, immune cell infiltration, fragmented muscle fibres and replacement of muscle fibres with connective tissue. Compared to their wild-type (wt) healthy littermates, male *mdx*^*5Cv*^ mice appeared similar in size and showed no significant reduction in their body weight (data not shown). After the mice were sacrificed, masseter muscles were dissected (Fig. 1a). Interestingly, at both 6 and 12 months of age, the weight of DMD masseter muscles was significantly lower than that of the wt mice (Fig. 1b,c).

Haematoxylin and eosin (H&E) staining of transverse sections from 3-, 6-, and 12-month-old *mdx*^*5Cv*^ mice showed areas of necrosis, a hallmark of dystrophic pathology (Fig. 1d), which was observed in the extensor digitorum longus (EDL) and soleus muscles (Supplementary Fig. S1a). Anti-IgM staining identified myofibers with compromised sarcolemmal integrity (Fig. 1e) in masseter from *mdx*^*5Cv*^ but not wt mice.

As an indicator of the progressive aspect of the condition, we would expect an initial increase in myotube necrosis followed by the replacement with scar tissue. Consistently, necrotic masseter muscle tissue, quantified from H&E staining as areas with infiltrating inflammatory cells and degenerating myofibers, was increased in 3-month-old *mdx*^*5Cv*^ mice compared to 6- and 12-month-old *mdx*^*5Cv*^ mice (*p* < 0.01) (Fig. 1d,f). In support of this finding, the percentage of anti-IgM positive myofibers was four times higher at 3 months in the masseter muscles of *mdx*^*5Cv*^ mice compared to 6- and 12-month-old *mdx*^*5Cv*^ mice (*p* < 0.0001) (Fig. 1e,g). We chose to compare the masseter muscles to the fast-twitch extensor digitorum longus (EDL) and slow-twitch soleus muscle as the pathology of the EDL and soleus muscles is well described in DMD mouse models^23,24^. Myonecrosis appears to be similar in masseter muscles compared to limb muscles for each respective age of the *mdx*^*5Cv*^ mice (Supplementary Fig. S1a,b).

**Fig. 2:**
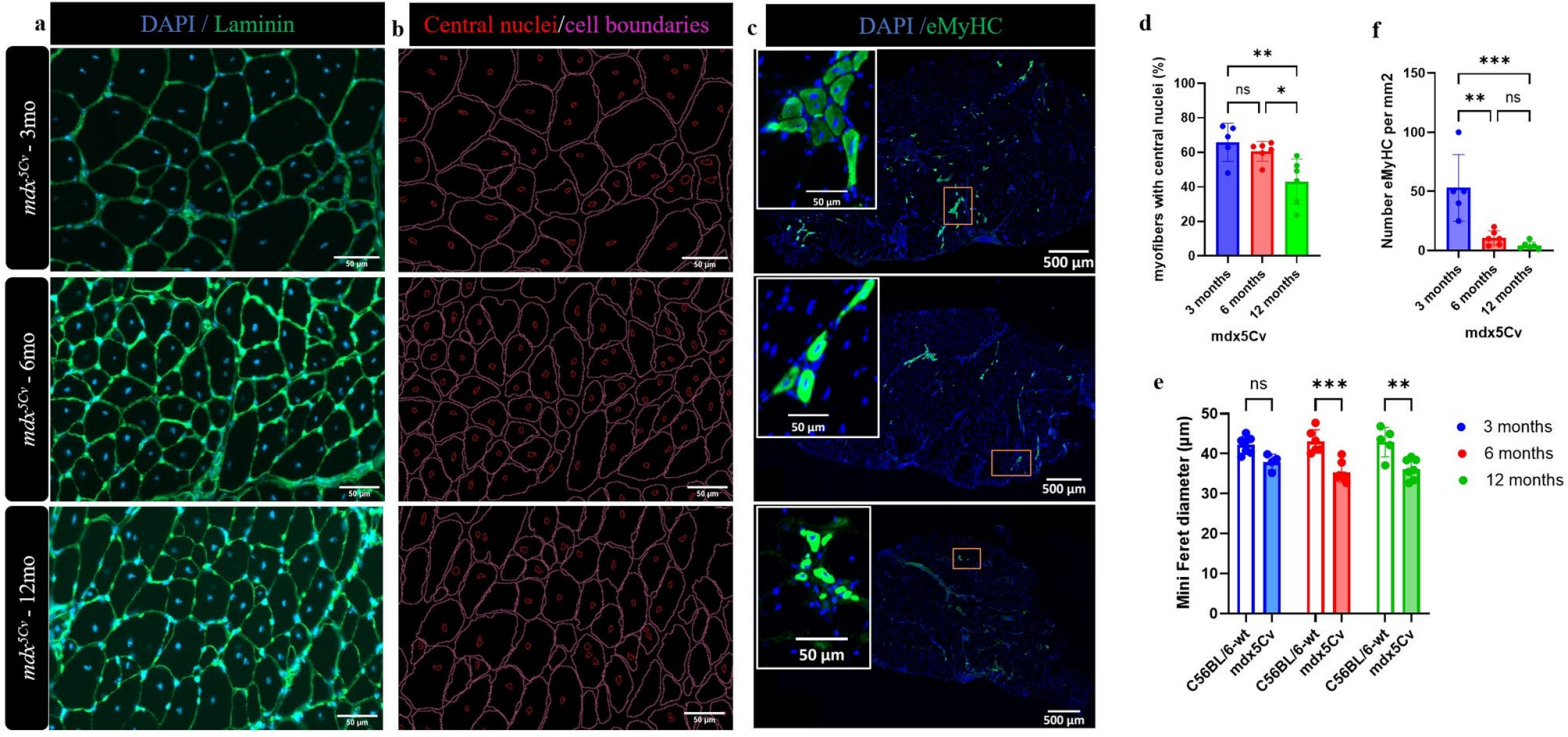
Regeneration in masseter muscles of *mdx*^*5Cv*^ mice. Representative fluorescence images of masseter muscles from 3-, 6- and 12-month-old *mdx*^*5Cv*^ mice stained with anti-laminin antibodies (green), and DAPI (blue) to reveal nuclei and cell boundaries, and their subsequent detection by Script CellP (**a**,**b**) (DOI: https://doi.org/10.5281/zenodo.14977977). Representative immunostaining of masseter muscles from 3-, 6- and 12-month-old *mdx*^*5Cv*^ mice stained with eMyHC (green) and DAPI (blue) (**c**). The percentage of muscle fibres containing central nuclei was determined in masseter muscle from 3-, 6- and 12-month-old wild type (wt) and *mdx*^*5Cv*^ mice (n = 5–7 mice/genotype) (**d**). The Feret diameter was determined in masseter muscles from 3-, 6- and 12-month-old wt and *mdx*^*5Cv*^ mice (n = 5–7) (**e**). The number of eMyHC per mm^2^ was determined in masseter muscles from 3-, 6- and 12-month-old wt and *mdx*^*5Cv*^ mice (n = 5–6 mice/genotype) (**f**). Pairwise analysis of the minimum Feret diameter between wt and *mdx*^*5Cv*^ mutant mice showed consistently smaller fibre diameters in the mutant condition. Data are presented as mean ± SD. Statistical analysis usied a one-way ANOVA with a post-hoc Tukey’s test (**d**,**f**) and two-way ANOVA with a post-hoc Sidak’s test (**e**). ns = not significant, *p < 0.05, **p < 0.01, ***p < 0.001, ****p < 0.0001 relative to age-matched wt mice.

Centrally nucleated fibres (CNF), a hallmark of muscle fibres that have undergone degeneration/regeneration cycles were not present in masseter muscles from wt mice. However, the proportion of muscle fibres with signs of CNF was approximately 60% in masseter muscles of *mdx*^*5Cv*^ mice at 3 months, 55% at 6 months, and 50% at 12 months (Fig. 2a,b,d). These proportions of CNF fibres were comparable to limb muscles of *mdx*^*5Cv*^ mice across all ages—3, 6, and 12 months—for the EDL, soleus, quadriceps, and tibialis anterior (TA) muscles (supplementary Fig. S2a,b).

### Masseter muscles of *mdx*^*5Cv*^ mice exhibit hallmarks of active dystrophic damage at least up to 12 months of age

We investigated the extent of active muscle regeneration across disease stages by quantifying fibres that express embryonic myosin heavy chain (eMyHC) on masseter muscle sections from *mdx*^*5Cv*^ mice at 3–12 months. Dystrophic masseter muscles at 3 months contained higher numbers of eMyHC-positive myofibers when compared to numbers at 6 months, with a decreasing number of eMyHC-positive myofibers upon disease progression at 12 months (Fig. 2c,f). We also stained for active regeneration (eMyHC) in *mdx*^*5Cv*^ masseter versus pathological limb muscles across all ages examined. We confirm that 60–80% of limb muscle fibres have centrally located nuclei, while showing few detectable active regenerating myotubes (Supplementary Fig. S3a,b). At any age examined here, eMyHC-positive fibres were much less abundant in any limb than in masseters muscles. This suggests an exacerbated dystrophic disease in masseter compared to locomotor muscles. This could suggest that this process occurs earlier in limb muscles compared to the masseter. Analysis of regeneration in limb muscles suggests a wave of injury/regeneration occurring before 3 months.

Subsequently, *mdx*^*5Cv*^ mice benefit from highly efficacious muscle regeneration; in contrast, progressive fibrosis, a major complication in DMD, occurs very late in *mdx*^*5Cv*^ mice. Subsequent to muscle fibre damage, the regeneration process gives rise to new myofibers (by detecting eMyHC protein), whose calibre is usually different from those of the original fibres (Fig. 2c). Myofiber size was evaluated using QuPath software by measuring the minimum Feret diameter (MFD) after laminin staining, revealing that the mean MFD in masseter muscles in *mdx*^*5Cv*^ mice was decreased compared with wt mice at all ages examined (Fig. 2a,b,e). Changes in fibre circularity have been proposed by others as a valuable metric when comparing muscles undergoing necrosis-regeneration, because fibres tend to gradually lose circularity over time. We observed a slight although non-significant decrease in fibre circularity over time in the masseter muscles of 6- and 12-month-old wt mice versus dystrophic mice (Supplementary Fig. S4).

### Characterization of collagen accumulation in the masseter muscles

**Fig. 3:**
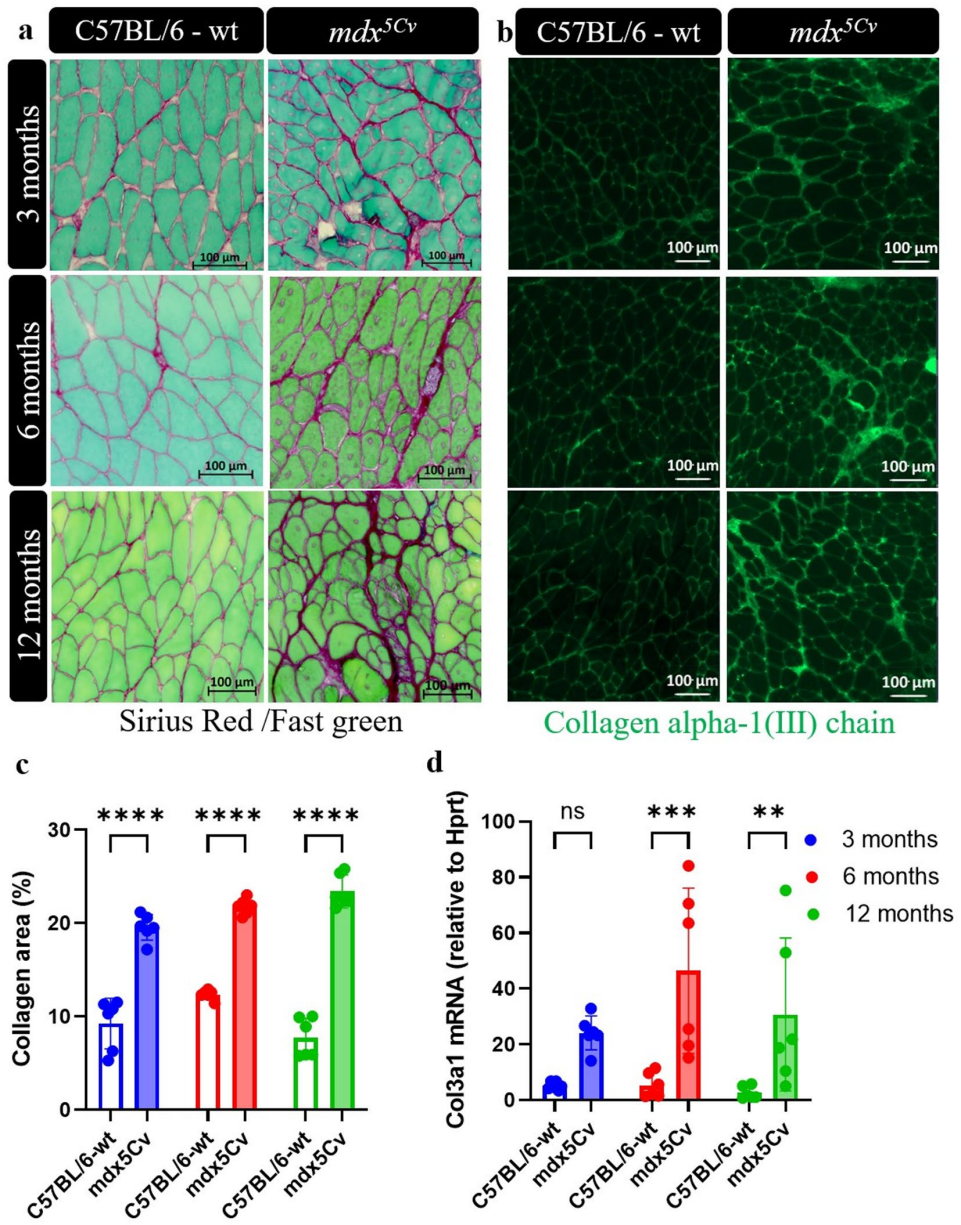
Collagen content is increased in masseter muscles of *mdx*^*5Cv*^ mice. Representative Sirius Red/Fast Green-stained sections of masseter muscle from a 3-, 6- and 12-month-old wild type (wt) and *mdx*^*5Cv*^ mice (n = 6 mice/genotype) (**a**). Masseter muscles were immunostained for collagen III (green) (**b**). The percentage of collagen area was determined based on Sirius red staining (Script: Find_Fibrosis; DOI: https://doi.org/10.5281/zenodo.14977977) (**c**). cDNA from the masseter muscles of *mdx*^*5Cv*^ mice was analysed by qRT-PCR for mRNA expression of *Col3a1* (n = 6) (**d**)*.* Data are presented as mean ± SD. Statistical analysis used a two-way ANOVA with a post-hoc Tukey’s multiple comparisons test (**b**–**d**). ns = not significant, *p < 0.05, **p < 0.01 relative to age-matched wt mice.

In order to evaluate the degree of myofiber fibrosis, we measured Sirius Red/Fast Green staining of collagen content at the different ages. In wild type mice, we found that the collagen content in masseter muscles was similarly low at all ages examined (Fig. 3a,c). The collagen content in masseter muscles of 6- and 12-month-old *mdx*^*5Cv*^ mice was significantly increased (Fig. 3a,b).

Sirius Red staining was conducted across all examined ages in the EDL and soleus muscles, and at 6 and 12 months in the quadriceps and TA muscles (Supplementary Fig. S5a,b), to evaluate ECM accumulation. Based on Qupath analysis, the results indicate that the masseter muscle displays a comparable degree of fibrosis compared to the EDL, soleus, and quadriceps muscles, and a slightly lower level compared to the TA muscles.

To further characterize the accumulation of collagen in masseter muscles, we stained sections with an antibody directed against the type III collagen alpha 1 chain (Col3α1), which represents a major fibrillary collagen constituent in the interstitial tissue of skeletal muscle^25^. Fluorescence microscope analysis revealed that collagen type III was present in young (3 months) and adult (6–12 months) wt mice. This accumulation over time was more pronounced in the masseter muscles of adult *mdx*^*5Cv*^ mice (Fig. 3c). At the mRNA levels, *Col3a1* expression was significantly increased at 6 months of age in *mdx*^*5Cv*^ mutant mice and remained elevated at least until 12 months of age (Fig. 3d).

### Analysis of the abundance of fibroadipogenic progenitors (FAPs) in masseter muscles of dystrophic mice

**Fig. 4:**
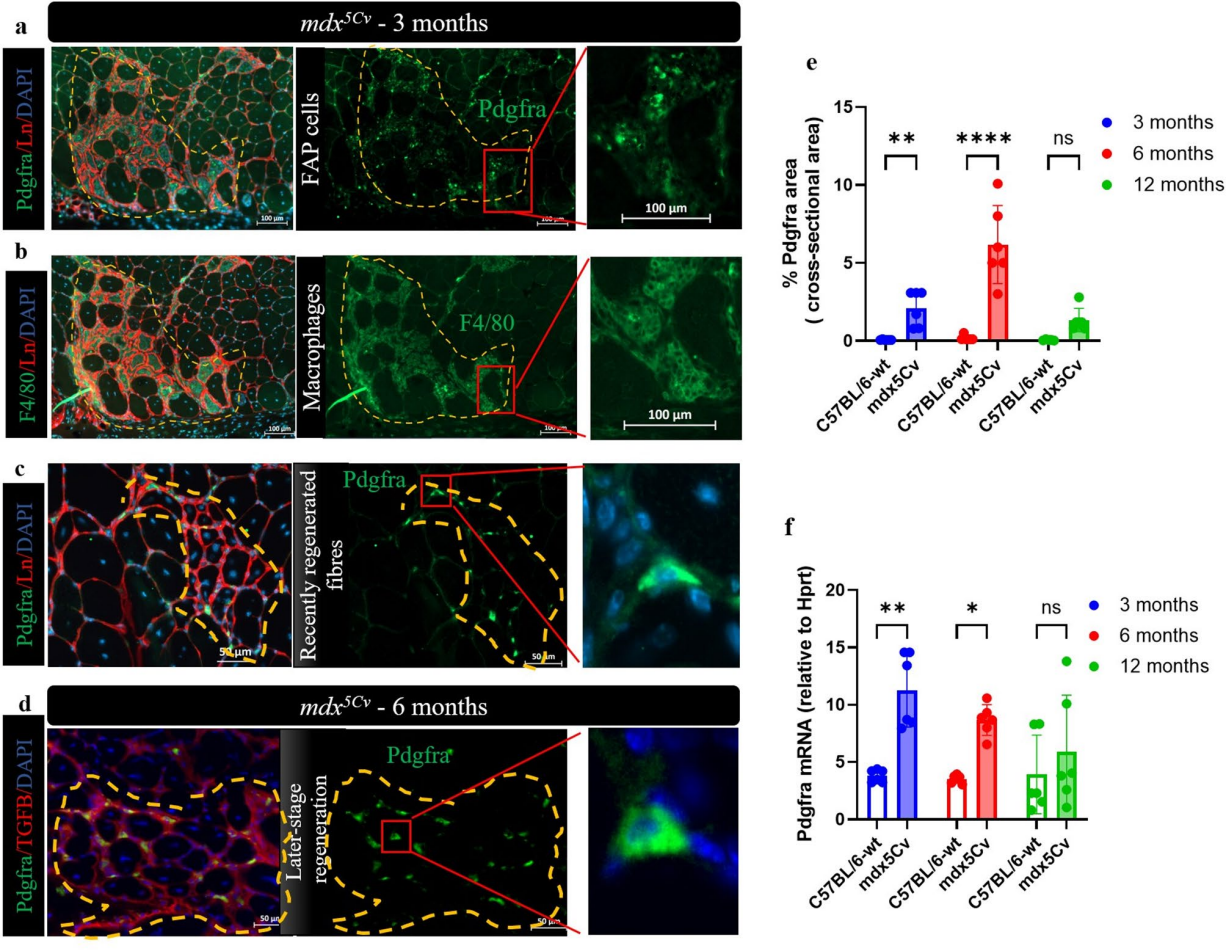
Fibro-adipogenic progenitors (FAPs) in masseter muscles of *mdx*^*5Cv*^ mice. Representative immunofluorescence of serial section of bona fide regenerating area in the masseter muscles of 3-month-old *mdx*^*5Cv*^ mice stained with anti-Pdgfra (green) and anti-F4/80 (green), laminin (red), and DAPI (blue) (**a**–**c**). Magnified images highlighting areas where macrophages (F4/80), FAPs (PDGFRA) and fibrotic areas (TGF-β3) are co-expressed (**a**–**d**). High-magnification images in the left panels indicate localization of PDGFRA + FAPs within foci containing immune cell (macrophages) (**a**,**b**), recently regenerating fibres (**c**) in 3-month-old *mdx*^*5Cv*^ mice and later stage regenerating area in 6-month-old *mdx*^*5Cv*^ mice (**d**). The percentage of FAP cells was quantified in masseter muscles of 3-, 6- and 12-month-old *mdx*^*5Cv*^ mice (**e**). Expression of *Pdgfra* mRNA in masseter muscles of 3-, 6- and 12-month-old *mdx*^*5Cv*^ mice was analysed by qRT-PCR (n = 6) (**f**). Data are presented as mean ± SD. Statistical analysis usied a two-way ANOVA with a post-hoc Tukey’s multiple comparisons test (**e**,**f**). ns = not significant, *p < 0.05, **p < 0.01, ****p < 0.0001 relative to age-matched wild type (wt) mice.

Muscle regeneration is regulated by many different cells, including immune cells and FAPs. The abundance of FAPs is dynamically regulated during muscle regeneration, and persistent FAP accumulation correlates with the severity of fibrosis in DMD muscle^26^. The localization and expression of FAPs in masseter muscles of *mdx*^*5Cv*^ mice was determined using antibodies for platelet-derived growth factor receptor-α (Pdgfra). Staining of serial sections with F4/80 revealed that FAP accumulation was observed in areas containing immune cells and identified as an intermediate regeneration stage (Fig. 4a,b). The macrophage staining with F4/80 in masseter muscles of 3-month-old *mdx*^*5Cv*^ mice was significantly increased and more pronounced in the masseter compared to limb muscles of *mdx*^*5Cv*^ mutant mice (Supplementary Fig. S6a,b).

We also observed the presence of FAPs in the vicinity of recently regenerated fibres, identified by small centrally located nuclei (Fig. 4c), as well as within areas of later-stage regeneration (Fig. 4d). The quantity of FAPs and the *Pdgfra* expression were significantly increased at 3 and 6 months of age in dystrophic mice, compared to the wt mice (Fig. 4e,f). Overall, these data suggest a crosstalk between FAPs and macrophages in dystrophic masseter muscles, as already suggested in other studies.

### Analysis of the decellularized ECM (dECM) in fibrotic foci in masseter muscles of dystrophic mice

**Fig. 5:**
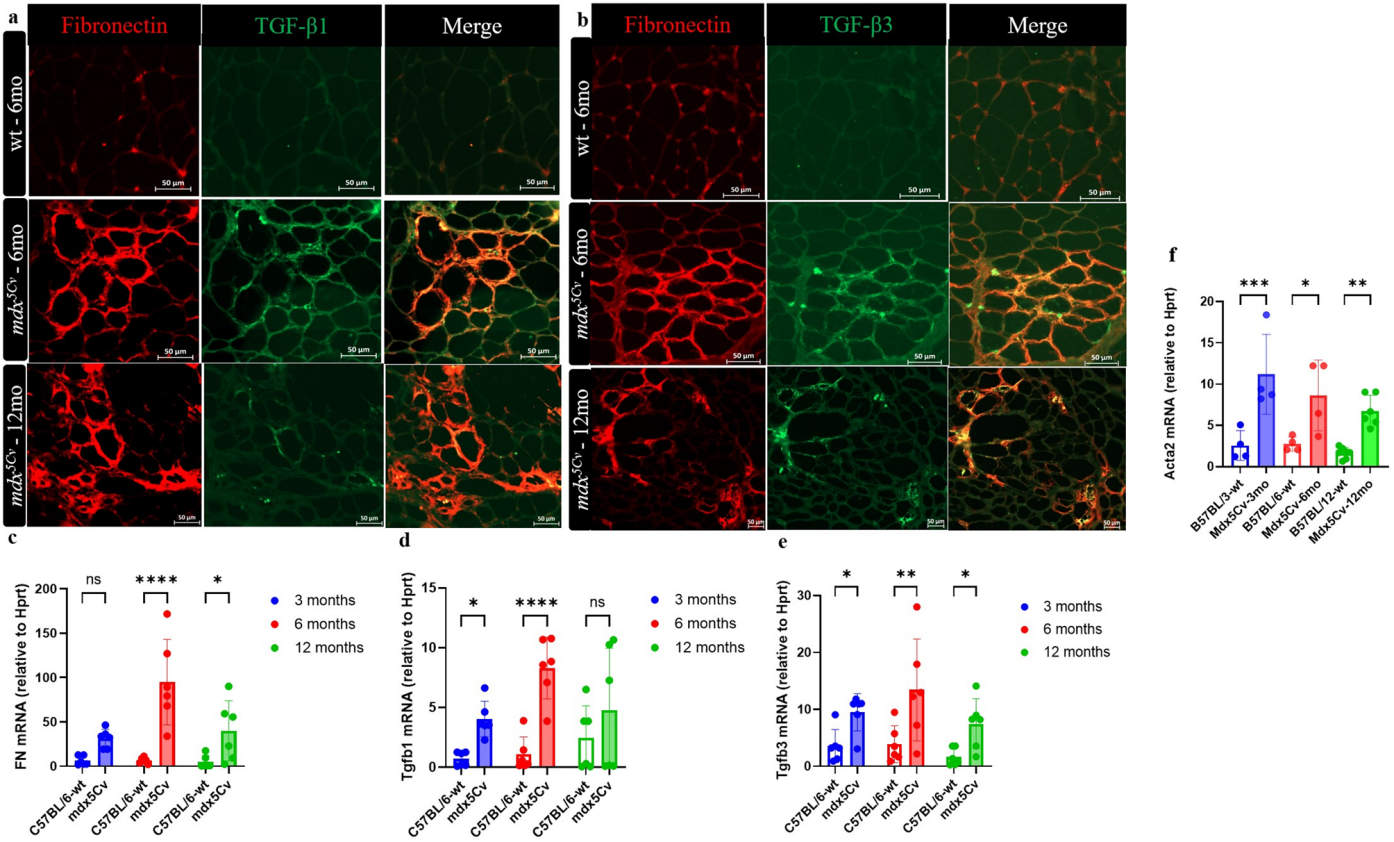
Deposition of latent TGF-β into the extracellular matrix of masseter muscles of *mdx*^*5Cv*^ mice. Representative immunofluorescence of “on slide” decellularized extracellular matrix (dECM) myoscaffolds were generated from *mdx*^*5Cv*^ masseter muscles. dECM myoscaffolds co-stained with anti-TGF-β1 (green) and fibronectin (red). Extracellular TGF-β1 and fibronectin expression were increased in fibrotic foci of *mdx*^*5Cv*^ (6-month-old) mice (**a**). dECM co-stained with anti-TGF-β3 (green) and fibronectin (red). Extracellular TGF-β3 and fibronectin expression were increased in fibrosis foci of *mdx*^*5Cv*^ (6 and 12 months) (**b**). Transcript abundance of *Fibronectin* (**c**)*, Tgf-β1* (**d**)*, Tgf-β3* (**e**) and *Acta2* (**f**) in *mdx*^*5Cv*^ mice was analysed by qRT-PCR (n = 6). Data are presented as mean ± SD. Statistical analysis used a two-way ANOVA with a post-hoc Tukey’s multiple comparisons test (**c**–**e**). ns = not significant, **p < 0.01, ****p < 0.0001 relative to age-matched wild type (wt) mice.

To determine accumulation of insoluble ECM fibres in masseter muscles of *mdx*^*5Cv*^ mice, an “on-slide” decellularization protocol (O’Brien et al. 2023) was performed. Masseter frozen sections were incubated in 1% Sodium Dodecyl Sulfate (SDS) and stained for collagen 1 to confirm removal of cellular material and retention of an intact ECM (data not shown). We observed an excess of TGF-β1 and TGF-β3 deposits throughout the ECM of masseter muscles of 6-month *mdx*^*5Cv*^ mice compared to wt mice (Fig. 5a,b). TGF-β is secreted into the environment as an inactive precursor, bound to the latency associated peptide (LAP). This molecule dimerizes and complexes with the latent TGF-β binding protein (LTBP) to be secreted and deposited within the ECM. Fibronectin (FN), a multifunctional glycoprotein that modifies the outcome of the phenotype of muscular dystrophy^27^ was also abundant in the dECM of *mdx*^*5Cv*^ myoscaffolds at 6 and 12 months (Fig. 5a,b). We could also observe a co-localisation of TGF-β1-FN (Fig. 5a merge) and TGF-β3-FN in fibrotic foci of masseter muscles (Fig. 5b merge). Interestingly, both *Tgf-β1* and *Tgf-β3* mRNA levels increased in masseter muscles of *mdx*^*5Cv*^ mice (Fig. 5d,e). Furthermore, FN expression was markedly upregulated at 6 and 12 months in masseters of *mdx*^*5Cv*^ mice when compared to wild type controls (Fig. 5c). It has also been shown that in the presence of TGF-β the upregulation of alpha-SMA is dependent on both SMAD2 and SMAD3^28^. *Acta2* expression was markedly upregulated across ages in masseter muscles of *mdx*^*5Cv*^ mice when compared to wt controls (Fig. 5f).

Overall, we observed increased expression of TGF-β3 in dystrophic masseter muscles across all ages examined, including at 3 months of age, prior to the observation of enhanced fibrosis. In contrast, TGF-β1 does not show a significant increase at 12 months. This suggests that TGF-β expressed at the early disease stage promotes later accumulation of fibrosis.

### TGF-β signalling is active in adult dystrophic masseter muscles

The dynamic expression profiles of TGF-β isoforms in adult dystrophic masseter muscles prompted us to evaluate the activity of TGF-β signalling at the cellular level. In order to detect cells exhibiting TGF-β signalling, we performed co-localisation staining for p-SMAD2 proteins and macrophages (F4/80) on cryosections of *mdx*^*5Cv*^ and wt masseter muscles.

**Fig. 6:**
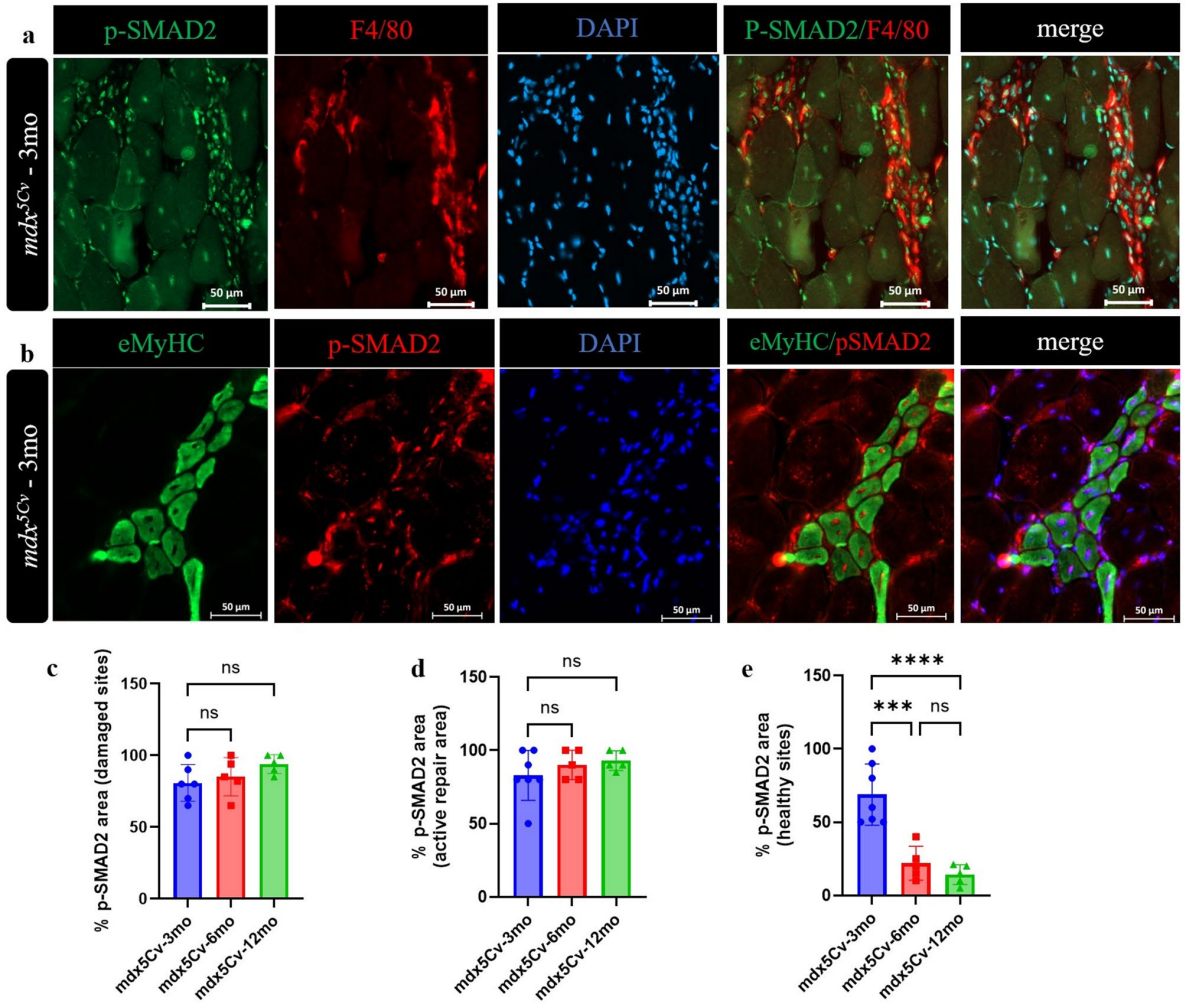
Expression of effectors of TGF-β signalling in dystrophic masseter muscles. Representative immunofluorescence of masseter muscle sections of 3-month-old *mdx*^*5Cv*^ mice stained with anti-F4/80 (red) and anti-p-SMAD2 (green), and DAPI (blue) (**a**). Representative immunofluorescence of bona fide regenerating area in masseter muscle sections of 3-month-old *mdx*^*5Cv*^ mice stained with anti-eMyHC (green) and anti-p-SMAD2 (red), and DAPI (blue) (**b**). The quantification of p-SMAD2 area locally normalized to areas of active damage (**c**), active repair (**d**) and in relatively healthy sites (e) was determined in masseter muscles from 3, 6- and 12-month-old wild type (wt) and *mdx*^*5Cv*^ mice (n = 5–6 mice/genotype). (Score_pSMAD2_cells; DOI: https://doi.org/10.5281/zenodo.14977977) (**e**). Data are presented as mean ± SD. Statistical analysis usde a one-way ANOVA with a post-hoc Tukey’s test (**e**) and two-way ANOVA with a post-hoc Sidak’s test (**d**). ****p < 0.0001 relative to age-matched wt mice.

In the muscle fibres of 3-month-old *mdx*^*5Cv*^ mice, the co-staining with p-SMAD2 and F4/80 clearly shows that the abundance and intensity of p-SMAD2 staining in damaged sites (inflamed) in *mdx*^*5Cv*^ muscle is greater at any age shown (Fig. 6a,c). We also examined co-staining with p-SMAD2 and F4/80 in EDL, quadriceps and TA (Supplementary Fig. S7). The results are consistent with those observed in the masseter muscle, showing that the abundance and intensity of p-SMAD2 staining in damaged (inflamed) regions of *mdx*^*5Cv*^ muscles are consistently higher across all ages compared to regenerating areas. We performed co-immunolocalization for p-SMAD2 proteins and regenerative markers (eMyHC) on cryosections of *mdx*^*5Cv*^* muscles,* with p-SMAD2 expression observed in centrally nucleated regenerating fibres (Fig. 6b,d). Dystrophic masseter muscles at 3 months contained higher numbers of p-SMAD2-positive myofibers in healthy sites when compared to numbers at 6 months, with a decreasing number upon disease progression at 12 months (Fig. 6e).

Together, these observations suggest that the abundance and intensity of p-SMAD2 staining in damaged sites (inflamed) in *mdx*^*5Cv*^ muscles is greater at any age shown, as compared to that in regenerating areas where inflammation is already resolving/resolved.

### Expression of RGD-binding Integrin-αv/β6 in dystrophic masseter muscles

**Fig. 7:**
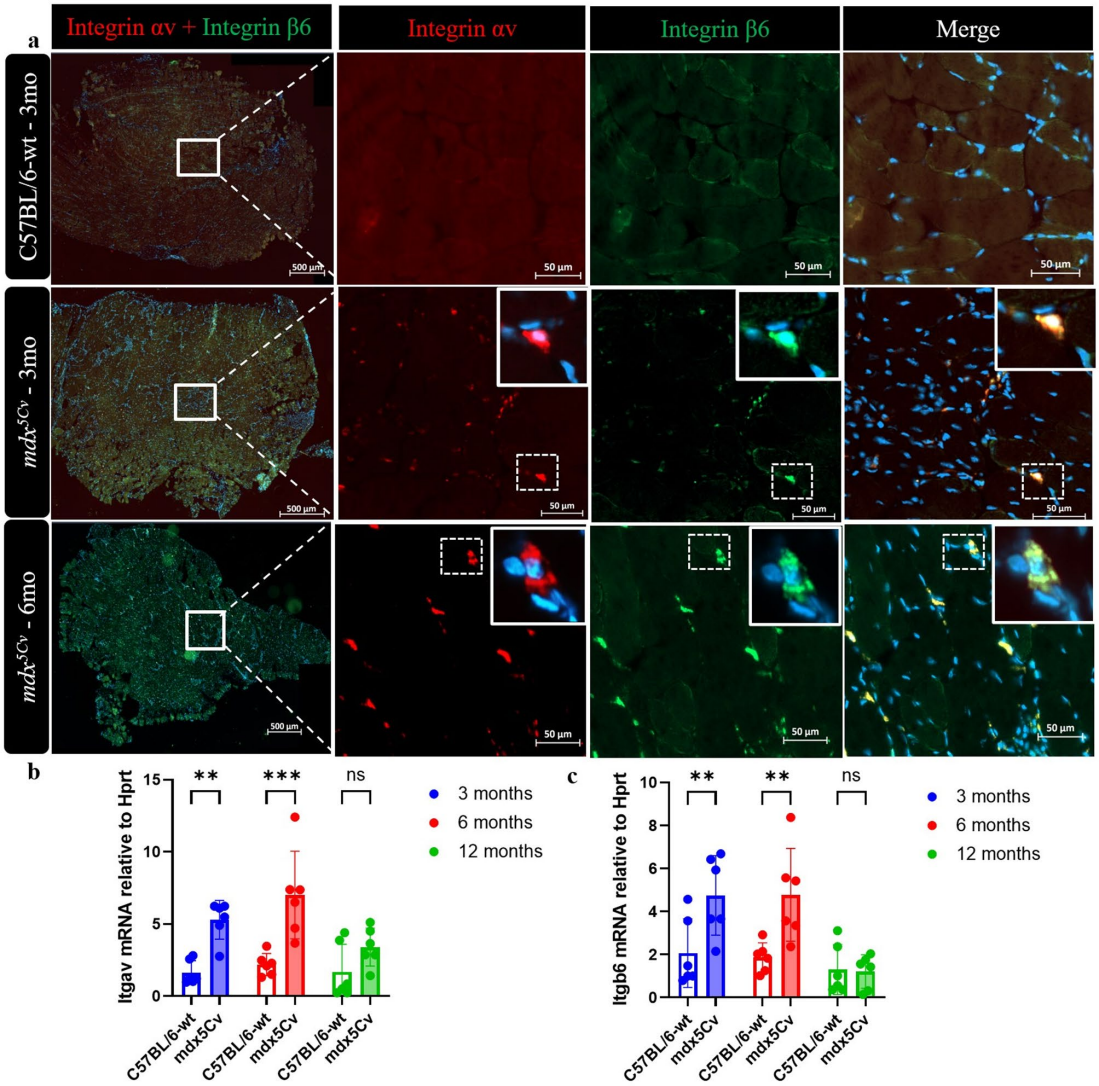
Expression of integrins required for TGF-β activation in dystrophic masseter muscles. Representative immunofluorescence of Integrin αv (red) and integrin β6 (green) staining on 3- and 6-month masseter muscle cryosections (**a**). cDNA from the masseter muscles of 3-, 6- and 12-month-old *mdx*^*5Cv*^ mice was analysed by qRT-PCR (n = 6) for mRNA expression of *Itgav* (**b**) and *Itgab6* (**c**). Data are presented as mean ± SD. Statistical analysis used a two-way ANOVA with a post-hoc Tukey’s test (**b**,**c**). ns = not significant, *p < 0.05, **p < 0.01 relative to age-matched wild type (wt) mice.

To understand the mechanism of TGF-β activation in masseter muscles, we first stained masseter sections with integrin subunits αv and β6 antibodies since the αvβ6 integrins are known to bind with high affinity to RGD motifs found in the TGF-β1 and TGF-β3 prodomain^29^. We detected αv and β6 integrin subunits in dystrophic *mdx*^*5Cv*^ masseter muscles from 3 to 6 months of age and not expressed in the wt control (Fig. 7a).

We detected αv and β6 integrin subunits in dystrophic *mdx*^*5Cv*^ masseter muscles from 3 to 6 months of age, compared to the wt control (Fig. 7a). Additionally, we observed colocalization of αv and β6 integrin, which is consistent with the elevated expression of integrin heterodimers αv/β6 in dystrophic masseter muscles.

At the mRNA level, *Itgav* expression increased at 3 months of age and persisted at high levels until 6 months in the dystrophic mice, compared to the wt mice (Fig. 7b). Similarly, *Itgb6* mRNA expression was also increased at 3 and 6 months in dystrophic masseter muscles (Fig. 7c), suggesting the increased expression of αvβ6 integrin subunits under dystrophic conditions. The pattern of αvβ6 expression mirrored that of TGF-β expression, indicating a direct role of αvβ6 integrin in the activation of latent TGF-β, to induce localised signalling in the regenerating tissue.

## Discussion

The pathological hallmarks of DMD include muscle necrosis, regeneration, inflammation, and fibrosis^4^. While clinical and pathological descriptions of DMD have primarily focused on limb muscles, respiratory muscles, and the heart, much remains to be learned about the manifestations of DMD within the orofacial muscles, including the masseter. To the best of our knowledge, this study is the first to characterize and compare masseter histopathology in the *mdx*^*5Cv*^ mouse model. We show that *mdx*^*5Cv*^ mice display a progressive dystrophic phenotype in the masseter muscles, resembling the pathology described previously in most limb muscles^21^. We have also shown an abnormal deposition of ECM including collagen, fibronectin and other ECM-associated signalling factors (TGF-β1 and TGF-β3), which can lead to the disruption of masseter architecture and ultimately orofacial dysfunction.

In this study, we propose histological and molecular clues regarding the dystrophic process in masseter muscles, and provide insights into the pathological accumulation of fibrotic tissue. The phenotype of the masseter muscle in *mdx*^*5Cv*^ mice observed in the current study aligns well with previous reports^30^. At birth, *mdx*^*5Cv*^ mice are asymptomatic and they develop normally until around weaning; then, within a few weeks, masseter muscles are affected with myonecrotic lesions. Subsequently and despite the ability of skeletal muscle to fully regenerate myofibers, *mdx*^*5Cv*^ mice show progressive fibrosis and accumulation of fibrous ECM in between individual muscle fibres within masseter muscles. Collectively, these data reveal that masseter muscles exhibit similar pathological features compared to the muscles commonly studied in preclinical research, such as prototypic leg muscles (EDL, TA, and soleus) and the heart^21,31^.

The mechanisms involved in the process of muscle degeneration in DMD have been extensively studied. It has been established that this process involves the participation of various cell types, including muscle stem cells (MuSC), FAPs, and inflammatory cells such as macrophages. These cells interact locally in what is referred to as the degenerative niche^32^. An increase in the population of FAPs is likely the primary cause of the expansion of the ECM. FAPs in the masseter muscle primarily originate from the neural crest, a structure of ectodermic origin, distinguishing them from the mesoderm-derived progenitors found in limb skeletal muscles. These crest-derived FAPs express markers such as PDGFRα and stem cell antigen (SCA). Moreover, these cells show a heightened tendency to undergo fibrosis, which is comparable to the fibrotic characteristics observed in ectoderm-derived tissues^33^. Interestingly, it has been demonstrated that FAPs are the main cells expressing ECM genes in regenerating skeletal muscles of both wt and *mdx*^*5Cv*^ mice^34^. We have observed a change in the composition of the ECM in *mdx*^*5Cv*^ mice characterized by an increase in collagen III, which has already been reported in skeletal muscle of *mdx* mice^35^. We have observed that accumulation of ECM occurs in foci spread all over the masseter tissue. Furthermore, an increase in the total number of macrophages and FAPs is observed, which is already evident from the earliest stages of fibrosis in the masseter muscles. FAPs and macrophages are localized in the same area, suggesting a durotactic attraction between FAPs and macrophages^36^. Alternatively, para-cellular TGF-β1-activation and signalling may require proximity^37^. However, under these conditions TGF-β1 is presented on the surface of macrophages in a GARP-dependent manner, and less associated to LTBP-deposited in the regenerating ECM scaffold. Once in proximity, fibroblasts and macrophages establish strong signalling, thereby creating a pro-fibrotic niche where pro-fibrotic genes produced by macrophages can be activated by myofibroblast contraction to regulate the progression of fibrosis. Nevertheless, and regarding the cell-surface and ECM-associated expression of latent forms of TGF-β1, further studies are needed to assess the interaction of immune and stromal cell and the direct influence by FAPs on the regenerative capacity of stem cells in dystrophic masseter muscles.

Transforming growth factor beta 1 (TGF-β1) is a well-studied cytokine within the TGF-β family, known for its crucial roles in muscle fibrosis development in muscular dystrophies^19^ In our study, we demonstrated that TGF-β-dependent signalling is enhanced in masseter muscles in *mdx*^*5Cv*^ mice. Furthermore, excessive TGF-β1 signalling is associated with fibrotic tissue stiffening^38^. TGF-β is secreted as an inactive, latent protein complex consisting of TGF-β and a noncovalently bound LAP, which requires further processing for activation. The latent TGF-β are stored in the ECM associated with LTBP’s, and tight regulation of its activation is critical to avoid uncontrolled TGF-β signalling^39^. Once in the extracellular environment, latent TGF-β1 must be activated to exert its effects and bind to its receptors^40^. Chazaud and colleagues demonstrated that latent TGF-β1 is activated by proteases produced by FAPs (Mmp13, Bmp1), and then released, active TGF-β1 then acts on FAPs and fibroblastic cells to promote fibrosis in the dystrophic muscle^41,42^. Specialized matrix proteins, such as certain fibronectin splice isoforms and thrombospondins have also been implicated in the activation of TGF-β in fibrotic tissues. The ED-A splice variant of fibronectin (ED-A FN) is markedly up-regulated in fibrotic tissues and contributes to TGF-β activation by immobilizing LTBPs into the matrix, thus localizing activatable TGF-β in the area of injury^43^. Fibronectin is also detectable at elevated levels in peripheral blood serum from patients with DMD^44^. These findings propose that latent TGF-β1 when activated plays a key role in the fibrosis observed in dystrophic muscles. In addition to its direct role in muscle, TGF-β1 has also been characterized as a central pro-fibrotic mediator in patients with DMD. Previous studies have shown that TGF-β1 levels are elevated in patients with DMD in both plasma and muscle, and the expression of TGF-β1 is correlated with fibrosis^17,45^. Furthermore, genome-wide mRNA profiling of DMD and control muscles indicates that the TGF-β pathway is highly induced in patients with DMD compared to controls^17,45^. Accordingly, in our study, we found enhanced levels of active Smad2 (as indicated by phosphorylated Smad2) and TGF-β target genes such as collagen and fibronectin, indicative of enhanced functional TGF-β signaling in masseter muscles of *mdx*^*5Cv*^ mice.

Another interesting change observed through disease progression is the increased expression of TGF-β3 at both the mRNA and protein levels, in dystrophic masseter muscles. In this study, *Tgf-β*1 and *Tgf-β*3 levels were similarly increased across disease progression. However, compared to wt mice, only *Tgf-β*3 levels remained significantly increased in the masseter muscles of dystrophic mice in more advanced fibrotic stages. This may be of high relevance to the pathogenesis, because although TGF-β1 has been extensively studied, specific roles for TGF-β3 have recently been proposed. Indeed, TGF-β3 has been shown to be implicated in the pathogenesis of fibrosis^46^. High *Tgf*-*β*3 levels have been shown to correlate with the severity of muscle fibrosis in dystrophic mice^47^. In DMD patients, TGF-β3 expression is markedly upregulated as compared to healthy controls, whereas TGF-β2 expression levels are comparable between DMD and control muscle (Geo dataset GDS214). Here, we show that mRNA and protein levels of *Tgf*-*β*3 are increased in severe dystrophic masseter muscles. Using immunofluorescence methods, we observed high amounts of latent *Tgf*-*β*3 in the extracellular matrix of masseter muscles in dystrophic mice. Single-cell RNA sequencing data demonstrates that in *mdx*^*5Cv*^ mice, *Tgf*-*β*3 is highly expressed in FAP cells in the diaphragm, while in contrast, *Tgf*-*β*1 is mostly expressed by macrophages^34^. We hypothesize that, similar to *mdx*^*5Cv*^ diaphragm, the elevated *Tgf*-*β*3 expression in dystrophic masseter muscles originates from increased numbers of FAP cells. While our study provides new aspects, and timing of TGF-β signaling in DMD, we are currently not in the position to individually evaluate the potentially differential contribution of the different TGF-β isoforms to DMD. Further studies are needed to elucidate the respective contributions of muscle fibres, macrophages, and FAPs to the TGF-β pathway.

Several studies report an anti-fibrotic function of TGF-β3^48,49^. Additionally, treatment with TGF-β3 has been observed to delay the onset and decrease the severity of radiation-induced pulmonary fibrosis in mice^50^. Despite major progress in characterizing the biochemical mechanisms of TGF-β activation, the pathomechanisms underlying the sustained activation of latent TGF-β3 in DMD fibrotic diseases remain incompletely understood. The latent TGF-β is stored in the ECM, and tight regulation of its activation is critical for function^27^. Interestingly, both LAP-TGF-β1 and -β3 contain the integrin binding motif RGDLXXL/I. αvβ6 and αvβ8 integrins, and are known to bind to this RGD motif with high affinities, mediating activation of TGF-β1 and TGF-β3 and outcome functions in vivo^29,51^. Since the expression of integrin αvβ6 was consistently increased in *mdx*^*5Cv*^ masseter muscles from 3 to 6 months old mice compared with the wt control (Fig. 7), this integrin could play an important role in the activation process of latent TGF-β1 and TGF-β3. Consistently, the pattern of αvβ6 expression mirrored that of p-SMAD2-positive cells, further proposing that this integrin is relevant in the DMD associated fibrosis.

In summary, this study investigated the process of muscle wasting in the masseter muscles at different stages of disease progression in dystrophic *mdx*^*5Cv*^ mice. Our results allow a longitudinal sequence of events to be proposed starting with degeneration, followed by regeneration, and fibrosis, which can lead to disruption of tissue architecture, masseter dysfunction and ultimately orofacial consequences. Our research offers additional insight into the expression of TGF-β isoforms and the way the orofacial muscles respond to dystrophic conditions and suggest roles for FAPs and TGF-β signalling in the pathogenesis. Understanding how the altered orofacial muscles impact overall function and bone growth, as well as its management, presents a significant challenge for dental professionals including orthodontists, as well as for the DMD community at large.

The current knowledge about the underlying pathophysiological mechanisms and empirical support for the management of DMD-related masticatory dysfunction remains neglected in clinical diagnosis and management guidelines and equally limited in preclinical research on muscular dystrophies. Rigorous development of preclinical models that recapitulate the dystrophic pathology in patients remain critical to better understand the pathological mechanisms involving orofacial muscles.

## Methods

### Animals and tissues

Masseter muscles used in the present study were obtained from untreated sedentary mice primarily dedicated to other experiments by LAN and OMD (unrelated data to be published elsewhere). All procedures involving mice complied with the Swiss Federal Law on Animal Welfare. Procedures were approved by the veterinary office of Geneva and authorized under the license numbers GE189/19, GE7220A and GE12420A. All methods were performed and reported in accordance with the ARRIVE guidelines and relevant regulations.

Mouse colonies were maintained at the animal facility of the Faculty of Medicine (University of Geneva, Switzerland) in conventional conditions. They were kept on a 12-h dark/12-h light cycle, under constant temperature (22 ± 1 °C) and humidity (50–60%). Mice were allowed unlimited access to food and water throughout the study.

The study focused on 3 age groups: young (3 months), adult (6 months), and mature adult (12 months). Each age group comprised 5–7 wild type (wt) mice (C57BL/6 J background) used as controls, and 5–7 *mdx*^*5Cv*^ dystrophic mice. Euthanasia was achieved under deep anaesthesia-analgesia: Animals were injected i.p. (1% body weight) with a mixture containing urethane (150 mg/mL), diazepam (0.5 mg/mL) and buprenorphine (10 μg/mL) in sterile saline (0.9% NaCl). After loss of consciousness was established, thoracotomy was performed, and heparin (1 μL/g body weight of a 3000 IU/mL solution) was injected into the heart. Death of the animal was achieved by collecting whole blood after sectioning of the aorta, dissecting the heart, and sectioning the spine prior to collecting the diaphragm. Masseter muscles from 36 male mice were dissected bilaterally and weighed. Additionally, the extensor digitorum longus (EDL) and soleus muscles from mice aged 3–12 months, as well as the quadriceps and tibialis anterior (TA) muscles from mice aged 6 and 12 months, were dissected for comparison with the masseter muscles.

### Histopathology

All muscles used were embedded in tragacanth gum, quickly frozen into liquid nitrogen-cooled 2-methylbutane and stored at − 80 °C until processed. Before sectioning, muscles were allowed to stand for 15–20 min in the chamber of a Leica cryostat set at − 20 °C. Sections of 10 μm were mounted on Superfrost Plus slides, which were stored at − 80 °C until staining. Before staining, slides were air-dried for 1 h, fixed in 4% paraformaldehyde for 10 min, and then stained with haematoxylin and eosin (H&E) according to conventional procedures. Skeletal myonecrosis was assessed in H&E stained tissue sections of transverse muscle as described in the Standard Operating Procedures (SOPs) defined by Treat-NMD.

For collagen staining, sections were fixed in methanol and stained for 30 min with Sirius Red/Fast Green solution (500 mg Direct Red 80 (Sigma 365548); 500 mg Fast Green FCF (Sigma F-7258) in 500 mL Picric acid solution (Sigma 925-40)), subsequently dehydrated, and mounted with Neo-mount. Collagen fibres appeared red, while the non-collagen proteins were green. Previous studies showed that the loss of muscle function is related to accumulation of ECM in the endomysium and perimysium, whereas fibrosis from the epimysium is not a relevant marker of the dystrophic disease.

### Immunofluorescence analysis

The sections were fixed in 4% paraformaldehyde and permeabilized for 10 min with 0.05% Triton in phosphate-buffered saline (PBS) before blocking non-specific binding sites in PBS containing 2% bovine serum albumin (BSA) for 45 min at room temperature (RT). Slides were then incubated with primary antibodies listed in Supplementary Table 1. All antibodies were diluted in 0.1% BSA and incubated overnight (o/n) at 4 °C, then exposed to Alexa Fluor-conjugated secondary antibodies for 45 min at RT. For embryonic myosin heavy chain (eMyHC) staining, PFA fixation was omitted. After rinsing, nuclei were stained with DAPI and slides mounted with Fluoromount-G Mounting Medium. Entire sections were imaged on a Zeiss AxioScan Z1 Automated Slide Scanner at 20× magnification and analysed with Zen software in conjunction with QuPath algorithms.

### QuPath analysis

For the evaluation of pathological features (fibrosis) as well as regeneration (percentage of central nuclei), the sections were scanned using a Zeiss Axio Scan.Z1 slide scanner and quantified with QuPath 0.5.1^52^ using custom scripts developed by the Bioimaging Core Facility, Faculty of Medicine, University of Geneva (DOI: 10.5281/zenodo.14977977). Briefly, Sirius Red and Fast Green chromogenic stains were separated using colour deconvolution^53^ leading to the creation of dedicated pseudo-channels. The Sirius Red channel was then thresholded to quantify collagen, which was normalized to tissue area. Previous studies showed that endomysium fibrosis is increased with DMD progression^54^. The percentage of centro-nucleated fibers was determined from entire sections, imaged on a Zeiss AxioScan Z1 slide scanner at 20× magnification and analysed with Zen software in conjunction with QuPath 0.4.2 using custom scripts developed by the same Bioimaging Core Facility (DOI: 10.5281/zenodo.14977977). First, a custom Cellpose model^55^ was trained to detect muscle fibres based on the laminin label with Alexa 488. Then, within each detected muscle fibre, nuclei labelled with DAPI were segmented using QuPath’s cell detection feature. We used circularity as an ROI measure to filter out longitudinally sectioned myofibers and elongated sectioned myofibers that may represent multiple myofibers (ROI < 0.3). Finally, the Euclidean distance from each nucleus to its respective muscle fibre border was computed, allowing for the characterization of muscle fibres based on the presence of internal nuclei. Morphological estimators, such as the minimum fibre diameter, were also reported.

### Decellularization and immunostaining of the ECM-associated secretome

Decellularization was performed as described by O’Brien et al. (2023). Frozen masseter muscles were cryosectioned at 10 µm and mounted on Superfrost Plus slides. The slides were stored at − 80 °C until decellularization. To decellularize the tissue, the slides were thawed at RT for 1 h and then placed in 1% UltraPure Sodium Dodecyl Sulfate (SDS) solution for 10–15 min. The slides were then rinsed in PBS (with calcium and magnesium) for 45 min, followed by a 30-min rinse in UltraPure Distilled H_2_O, and then an additional 45-min wash in PBS. Decellularized muscle cryosections were fixed in 4% paraformaldehyde, rinsed, and incubated in a blocking buffer (PBS containing 1% BSA and 10% foetal bovine serum) at RT for 1 h. The scaffolds were incubated in primary antibodies overnight at 4 °C. Alexa Fluor 488-conjugated goat anti-mouse IgG was used at 1:500 for 1 h at RT. Images were acquired on an Axio Scan Z1 microscope with identical exposure settings across genotypes.

### Gene expression and quantification

RNA extraction was performed on 200 μm-thick cryosections. Total RNA was extracted using NucleoZOL (Macherey-nagel, Duren, Germany). cDNA was synthesized from 1 μg RNA using qScript™ cDNA SuperMix (Quantabio order number 95048-025). qPCR was performed using the StepOne and StepOnePlus Real-Time PCR Systems (Applied Biosystems, California, USA) and PowerUp™ SYBR™ Green Master Mix (Applied Biosystems, A25741). To determine the changes in steady-state, mRNA was quantified relative to a standard curve generated with serial dilutions of a control cDNA preparation and normalized against Hprt mRNA, a housekeeping gene whose expression is stable in dystrophic mice based on geNorm methods. Raw Ct values were imported into Excel, and normalization factors and fold changes were calculated using the geNorm method^56^. All experiments were repeated at least two times by the same operator on two different occasions. Primers used for RT-qPCR are listed in Supplementary Table 2.

### Statistical analysis

Statistical analyses were performed using GraphPad PRISM (version 9.3.0 463). Two-way ANOVA tests were used to detect differences between the dystrophic and wild type mice, at different ages, followed by post-hoc Tukey’s multiple comparison test or a post-hoc Šídák's multiple comparisons test. Non-parametric Kolmogorov–Smirnov tests were used to analyse the distribution of fibre circularity. *p* values of < 0.05 were considered statistically significant, with the following thresholds applied: *p < 0.05, **p < 0.01, ***p < 0.001, and ****p < 0.0001. Software used in this paper are listed in Supplementary Table 3.

## Supplementary Information

Below is the link to the electronic supplementary material.

Supplementary Material 1

## Data Availability

Raw data (from immunochemistry and gene expression quantifications) were generated at Division of orthodontics, University clinics of dental medicine, Faculty of Medicine, University of Geneva, Switzerland. Derived data supporting the findings of this study are available from the corresponding authors A.A.L upon reasonable request. Custom scripts were developed by the Bioimaging Core Facility, Faculty of Medicine, University of Geneva and stored in Zenodo (DOI: 10.5281/zenodo.14977977).
