# Supplementary material for: Persistent tissue regeneration and transforming growth factor-β induced fibrosis in the masseter muscle of mdx5Cv mice
Source: Sci Rep. 2025 Sep 29;15:33503. doi: 10.1038/s41598-025-17154-3 (PMC12480963; doi:10.1038/s41598-025-17154-3)

**Supplementary material**

**Persistent tissue regeneration and transforming growth factor-β induced fibrosis in the masseter muscle of *mdx^5Cv^* mice**

**Running title: TGF-β signalling in dystrophic masseter muscles**

Aouatef Ait-Lounis, Laurence A. Neff, Stavros Kiliaridis, Bernhard Wehrle-Haller, Olivier M. Dorchies, Gregory S. Antonarakis

Supplementary Table 1: Antibodies used as markers for different cells and cellular processes

Supplementary Table 2: Primers used for quantitative reverse transcriptase PCR (qRT-PCR)

Supplementary Table 3: Software and databases used in this paper

Supplementary Fig 1: Representative haematoxylin & eosin-stained sections of EDL and soleus (SOL) muscles from 3-month-old mice, and EDL, SOL, quadriceps, and tibialis anterior muscles from 6- and 12-month-old *mdx^5Cv^* mutant mice (n = 6) are shown in (a). Sections were quantified to determine the percentage of total necrotic myofibers in the masseter (MAS), EDL, and SOL muscles of dystrophic 3-month-old mice, as indicated by infiltrating inflammatory cells (blue arrows) in (b).

Supplementary Fig 2: Representative fluorescence images of EDL and soleus (SOL) muscles from 3-month-old mice, and of EDL, SOL, quadriceps (QC), and tibialis anterior (TA) muscles from 6- and 12-month-old *mdx^5Cv^* mutant mice (n = 6) are shown in (a). Tissues were stained with anti-laminin antibodies (red) to visualize muscle fibre boundaries and with DAPI (blue) to label nuclei. Images were analysed using Script CellP. The percentage of muscle fibres containing centrally located nuclei was quantified in the masseter, EDL, and SOL muscles at 3, 6, and 12 months of age, and in the QC and TA muscles at 6 and 12 months (n = 2–6 mice per genotype).

Supplementary Fig 3: Representative fluorescence images of EDL and soleus (SOL) muscles from 3-month-old mice, and of EDL, SOL, quadriceps (Quad), and tibialis anterior (TA) muscles from 6- and 12-month-old *mdx^5Cv^* mutant mice (n = 6) are shown in (a). Tissues were stained with anti-laminin antibodies (red) to visualize muscle fibre boundaries, DAPI (blue) to label nuclei and eMyHC in green (white arrows). The number of eMyHC per mm^2^ was quantified in the masseter (mas), EDL, and SOL muscles at 3, 6, and 12 months of age, and in the Quad and TA muscles at 6 and 12 months (n = 2–6 mice per genotype).

Supplementary Fig 4: Distribution of fibre circularity in masseter muscles from 3-, 6-, and 12-month-old *mdx^5Cv^* mutant mice. Muscles were stained with anti-laminin antibodies to visualize cell boundaries, and fibre circularity was subsequently analysed using Script CellP (DOI:10.5281/zenodo.14977977).

Supplementary Fig 5: Representative Sirius Red/Fast, Green-stained sections of EDL and soleus (SOL) muscles from 3-month-old mice, and of EDL, SOL, quadriceps (QC), and tibialis anterior (TA) muscles from 6- and 12-month-old *mdx^5Cv^* mutant mice are shown in (a). Sections were quantified to determine the percentage of total necrotic myofibers (b). The percentage of fibrotic area was assessed based on Sirius Red staining (Script: Find_Fibrosis; DOI:10.5281/zenodo.14977977).

Supplementary Fig 6: Representative immunofluorescence images of masseter (MAS), EDL, and soleus (SOL) muscle sections from 3-month-old *mdx^5Cv^* mice stained with anti-F4/80 (green), laminin (red), and DAPI (blue) are shown in (a). The percentage of macrophages was quantified in the masseter and limb muscles of 3-, 6-, and 12-month-old *mdx^5Cv^* mice (b).

Supplementary Fig 7: Representative immunofluorescence images of limb muscle sections (EDL, quadriceps, and tibialis anterior) from 3- and 6-month-old *mdx^5Cv^* mice stained with anti-F4/80 (red), anti-p-SMAD2 (green), and DAPI (blue).

Supplementary Table 1

| Antibodies | Clone | Work dilution | Cell target | Source | Identifier |
| --- | --- | --- | --- | --- | --- |
| TGF-β1 | 3C11 | 1/100 | Pro-fibrotic cytokine | Santa Cruz Biotechnology | Cat. sc-130348 RRID: AB_1567351 |
| TGF-β3 | G-9 | 1/100 | Pro-fibrotic cytokine | Santa Cruz Biotechnology | Cat. sc-166833 RRID:AB_2303246 |
| Pdgfra (cd140a) | AP5 | 1/100 | FAPs | Thermo Fisher Scientific | Cat. 14-1401-82 RRID:AB_467491 |
| F4/80 | ci-a3-1 | 1/40 | Pan-macrophage | Abcam | Cat. ab6640 RRID:AB_1140040 |
| Fibronectin | C1801 | 1/1000 | Extracellular matrix | Donation by B. Wehrle-Haller | - |
| Laminin | - | 1/1000 | Muscle fibre basal lamina | Abcam | Cat. ab11575 RRID:AB_298179 |
| Collagen III | FH-7A | 1/200 | Extracellular matrix | Abcam | Cat. ab6310 RRID:AB_305413 |
| SMAD2 (phosphor Ser465/Ser467) | - | 1/100 | TGF-β signalling | GeneTex | Cat. GTX133614 RRID:AB_2887051 |
| DAPI | - | Ready to use | Cell nuclei | Vector Laboratories | Cat. H-1500  RRID:AB_2336788 |
| CD51 (Integrin alpha V) | RMV-7 | 1/100 | TGF-β activation | Thermo Fisher Scientific | Cat. 14-0512-82  RRID:AB_467296 |
| ITGB6 Polyclonal | PA5 | 1/100 | TGF-β activation | Thermo Fisher Scientific | Cat. PA5-47309  RRID:AB_2576254 |
| Myh3 (eMyHC) | F1.652 | 1/100 | marker of newly formed fibres | DSHB | Cat# F1.652, RRID:AB_528358 |

Supplementary Table 2

| Gene Name | Sequence (5' -> 3') |
| --- | --- |
| *Hprt-fw* | AAG CTT GCT GGT GAA AAG GA |
| *Hprt-rv* | TTG CGC TCA TCT TAG GCT TT |
| *Col3a1-fw* | GCC CAC AGC CTT CTA CAC |
| *Col3a1-rv* | CCA GGG TCA CCA TTT CTC |
| *Pdgfra -fw* | GTC CAG GTG AGG TTA GAG G |
| *Pdgfra-rv* | CAC GGA GAA CAA AGA C |
| *Tgfβ1-fw* | CAT CCA TGA CAT GAA CCG GC |
| *Tgfβ1-rv* | GAA GTT GGC ATG GTA GCC CT |
| *Tgfβ3-fw* | AGA CAC AAC CCA TAG CAC GG |
| *Tgfβ3-rv* | CCT CAG CTG CAC TTA CAC GA |
| *Itgav-fw* | GTG TGA GGA ACT GGT CGC CTA T |
| *Itgav-rv* | CCG TTC TCT GGT CCA ACC GAT A |
| *Itgb6-fw* | TTG CTC AAA GCT TGG TT |
| *Itgb6-rv* | ATC CAT AGA GGC GGA GAG GT |
| *Fn1-fw* | AAA CTT GCA TCT GGA GGC AAA CCC |
| *Fn1-Rv* | AGC TCT GAT CAG CAT GGA CCA CTT |
| *Acta2-fw* | TCA GCG CCT CCA GTT CCT |
| *Acta2-Rv* | AAA AAC CAC GAG TAA CAA ATC AA |

Supplementary Table 3

| ZEN black software | Carl Zeiss Microscopy | <https://www.zeiss.ch/> |
| --- | --- | --- |
| QuPath software | Oxford Instruments | <https://qupath.github.io/> |
| ImageJ software | NIH | <https://imagej.net/ij/> |
| Antibody Registry beta | [dkNET's Resource Reports - Antibodies](https://dknet.org/data/source/nif-0000-07730-1/search) | <https://www.antibodyregistry.org/> |
| GraphPad Prism software version 9.3.0 | GraphPad Software | <https://www.graphpad.com/> |
| TREAT-NMD  Standard operating procedures (SOP library) | European neuromuscular network | https://www.treat-nmd.org/resources-and-support/sop-library/mdx-mouse-dmd/ |
| UCSC genome browser ([In-Silico PCR](https://genome.ucsc.edu/cgi-bin/hgPcr)) | NIH | https://genome.ucsc.edu/cgi-bin/hgPcr |
| Zenodo repository | CERN | https://zenodo.org/ |

Supplementary Figure 1


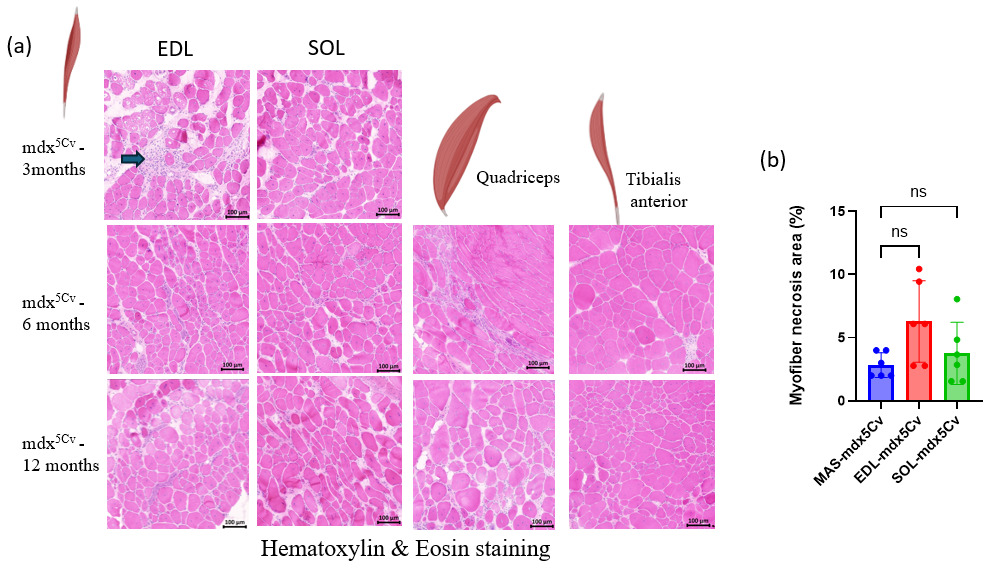


Supplementary Figure 2


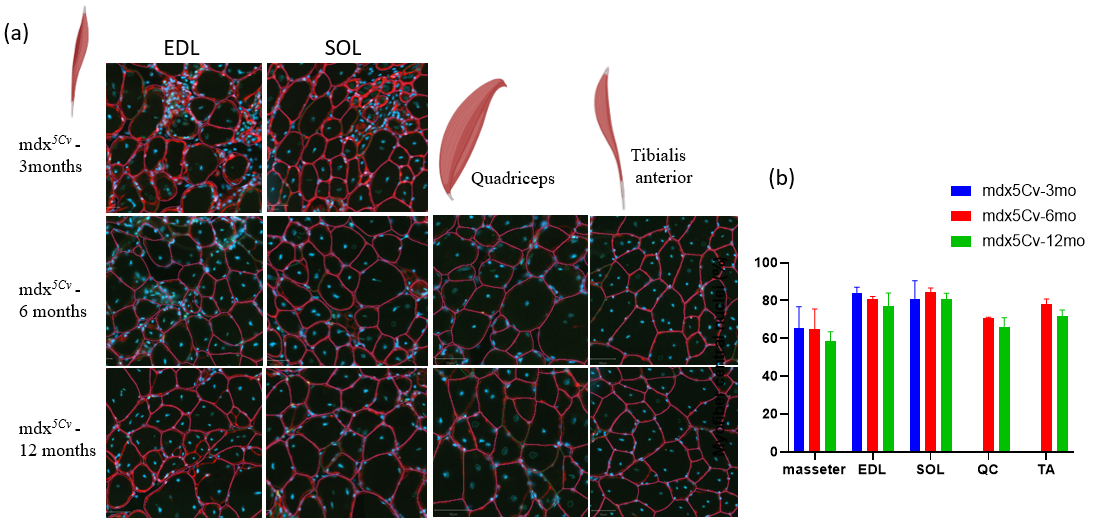


Supplementary Figure 3


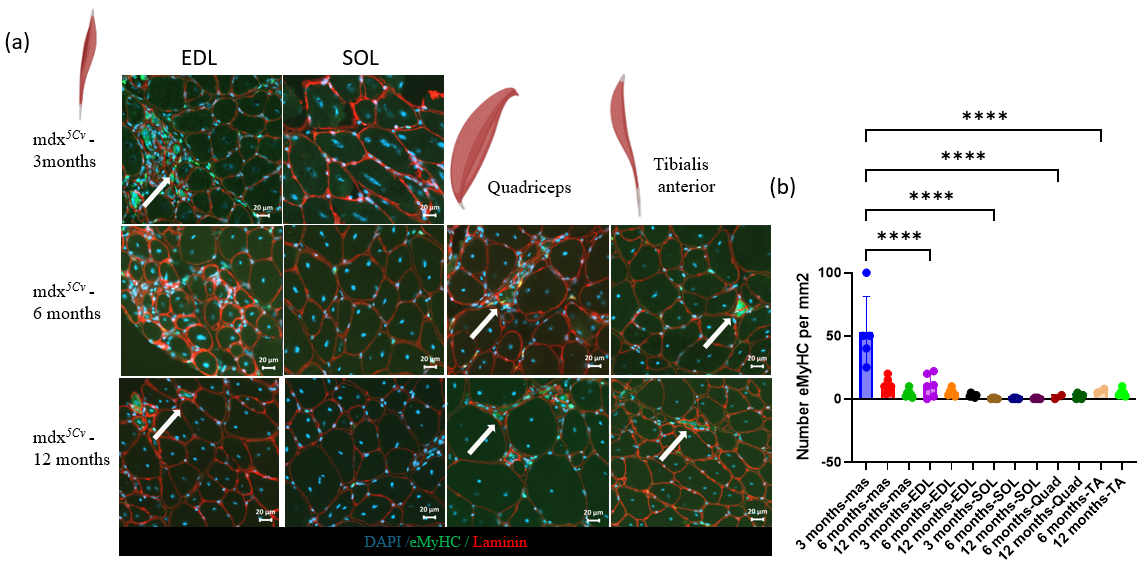


Supplementary Figure 4


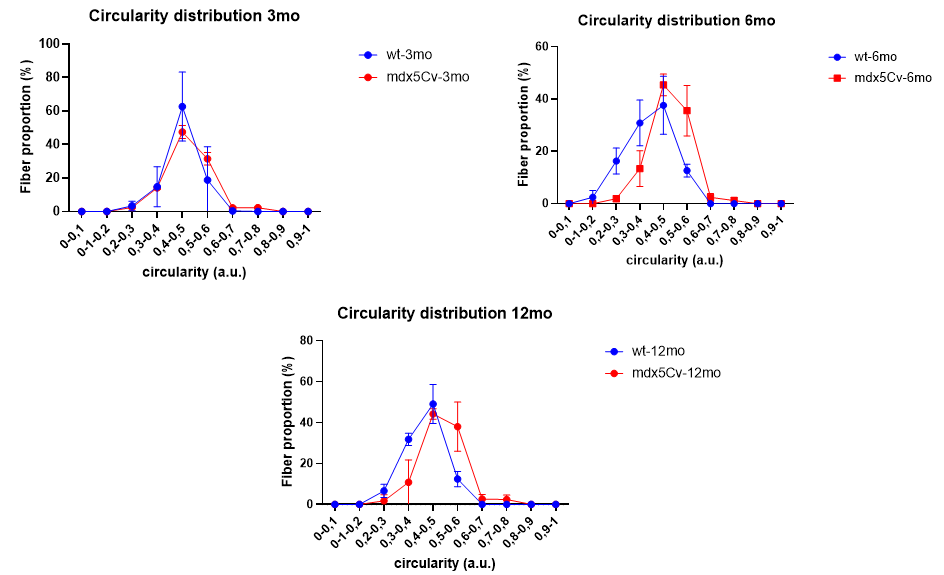


Supplementary Figure 5


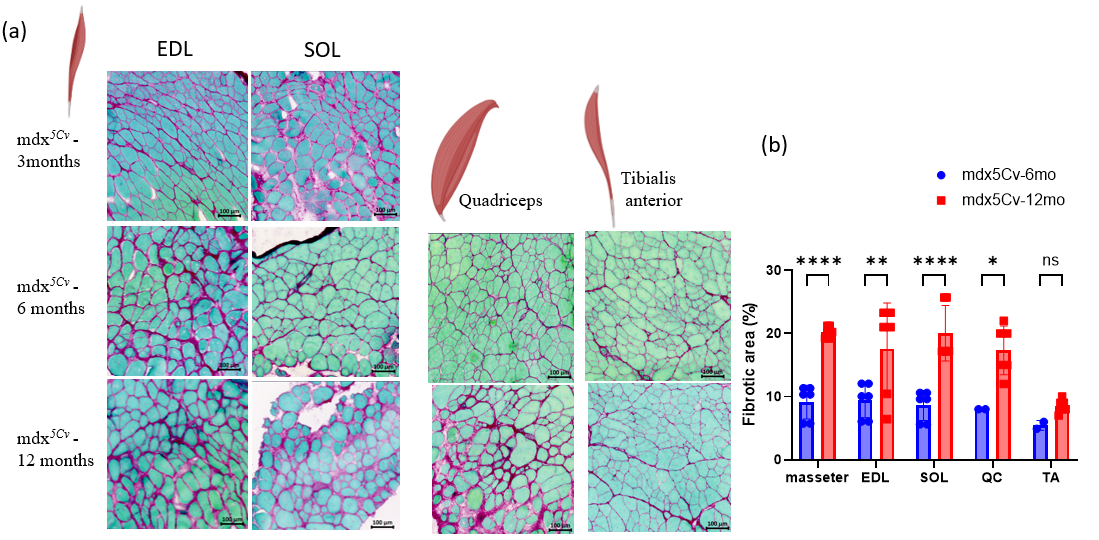


Supplementary Figure 6


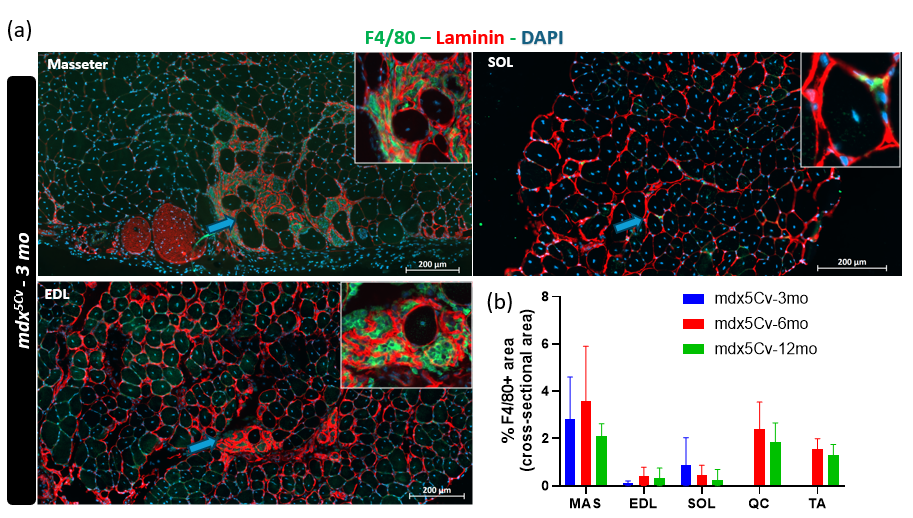


Supplementary Figure 7


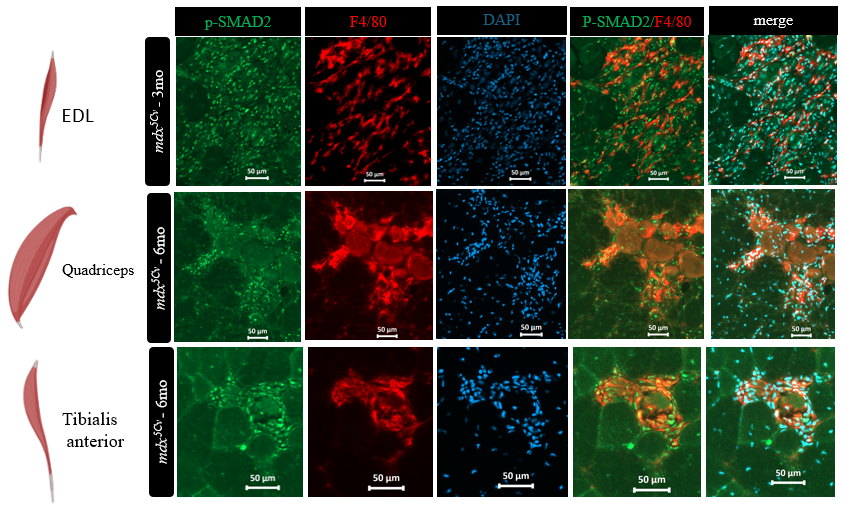

Supplement: Supplementary file 1 — Supplementary Material 1 [file 41598_2025_17154_MOESM1_ESM.docx]
